# Construction of a high-density genetic linkage map and QTL mapping of growth and cold tolerance traits in *Takifugu fasciatus*

**DOI:** 10.1186/s12864-023-09740-4

**Published:** 2023-10-27

**Authors:** Ying Zhang, Jie Li, Peng Chu, Ruhua Shang, Shaowu Yin, Tao Wang

**Affiliations:** College of Marine Science and Engineering, Nanjing Normal University, Nanjing, 210023 Jiangsu China

**Keywords:** *Takifugu fasciatus*, Genetic linkage map, QTL mapping, Growth and cold tolerance traits

## Abstract

*Takifugu fasciatus* is an aquaculture species with high economic value. In recent years, problems such as environmental pollution and inbreeding have caused a serious decline in *T. fasciatus* germplasm resources. In this study, a high-density genetic linkage map was constructed by whole-genome resequencing. The map consists of 4891 bin markers distributed across 22 linkage groups (LGs), with a total genetic coverage of 2381.353 cM and a mean density of 0.535 cM. Quantitative trait locus (QTL) localization analysis showed that a total of 19 QTLs associated with growth traits of *T. fasciatus* in the genome-wide significance threshold range, distributed on 11 LGs. In addition, 11 QTLs associated with cold tolerance traits were identified, each scattered on a different LG. Furthermore, we used QTL localization analysis to screen out three candidate genes (*IGF1*, *IGF2*, *ADGRB*) related to growth in *T. fasciatus*. Meanwhile, we screened three candidate genes (*HSP90*, *HSP70*, and *HMGB1*) related to *T. fasciatus* cold tolerance. Our study can provide a theoretical basis for the selection and breeding of cold-tolerant or fast-growing *T. fasciatus.*

**Supplementary Information:**

The online version contains supplementary material available at 10.1186/s12864-023-09740-4.

## Introduction

*Takifugu fasciatus* is an aquaculture species with high economic value, that is widely distributed in the Sea of Japan, the East China Sea, and the Yellow Sea [1, 2]. It is very popular with consumers because of its delicious meat and high nutritional value, and the market demand for this species is constantly growing, leading to a gradual increase in its production [3]. As the scale of *T. fasciatus* culture has expanded, a series of problems has emerged, such as the degradation of germplasm resources caused by inbreeding and overfishing of wild resources, specifically in the form of reductions in cold tolerance and growth rates, seriously limiting the development of the *T. fasciatus* aquaculture industry. Good production and superior traits of aquaculture species are important indicators of development in the aquaculture industry. Improving the growth outcomes and optimizing the growth traits of farmed fish can not only shorten the breeding cycle and save on breeding costs but also increase production [4]. Moreover, because *T. fasciatus* is a warm water fish, whose ideal water temperature range is currently limited 23–32 °C [1], improving its cold tolerance can have high economic benefits [5, 6]. Many efforts have been made in genome-wide studies to promote the development of molecular assisted breeding for *T. fasciatus* aquacultures*.* In 2006*,* Ma et al. [7] used the simple sequence repeat (SSR) and bulk segregant analysis (BSA) technologies in combination to screen two loci, fms15 and fms75, which were significantly correlated with the growth of *T. fasciatus.* In 2020, Kang et al. [8] published a chromosome-level reference genome for *T. fasciatus* that provides an important genomic resources for growth and cold tolerance traits selection. However, several certain limitations related to several specific traits continue to hinder the development of traditional phenotype-based selection breeding techniques, such as the lack of large-scale genomic resources and markers closely associated with growth and cold-tolerance related traits. Therefore, it is imperative to carry out research on the germplasm resources and genetic improvement of *T. fasciatus.*

At present, marker-assisted selection (MAS) is a strategy that can reduce the time it takes to breed new varieties and is considered effective and is widely used in genetic improvement methods such as selective breeding [9]. The construction of a genetic linkage map is the first step in the development of aquaculture animal genome research. High-density genetic linkage maps are an indispensable tool of genomics and genetics, providing an important basis for quantitative trait loci (QTL) mapping in MAS in species, such as *Scophthalmus maximus* [10], *Cyprinus carpio haematopterus* [11] and *Mylopharyngodon piceus* [12]. The *T. fasciatus* genome size is approximately 381 Mb, and no genetic linkage maps for *T. fasciatus* have yet been reported [8]. The whole-genome resequencing (WGR) technique is a very effective and suitable MAS method for identifying breeding programmes to target the specific characteristics of *T. fasciatus*. The whole-genome sequencing of different individuals of a species for which a reference genome sequence is available, and the individual sequencing data are compared with the reference genome sequence for differential analysis [13, 14]. WGR can be used to conduct comprehensive scans, detect genome variation information, and uncover numerous variation loci at one time with high accuracy and reproducibility. The identification of SNPs based on WGR is widely used in aquatic biology, such as in studies of *Seriola dumerili* [15], *Oreochromis* [16] and *Oncorhynchus mykiss* [17]*.*

In this study, a high-resolution genetic linkage map was established using WGR techniques, as well as fine mapping of economic QTL traits, including 3 growth-related traits and 1 temperature-related trait. Moreover, a number of SNP loci related to growth and low temperature tolerance were identified by QTL localization, and eight candidate genes were selected by comparing genome-wide information. The expression of the six candidate genes was analysed by real-time quantitative PCR (qRT-PCR) with the aim of providing a theoretical basis for the molecular breeding of traits related to growth and low temperature tolerance in *T. fasciatus*. The data obtained from the study will provide a strong basis for the application of MAS in *T. fasciatus*.

## Results

### Phenotypic trait characteristics

**Fig. 1:**
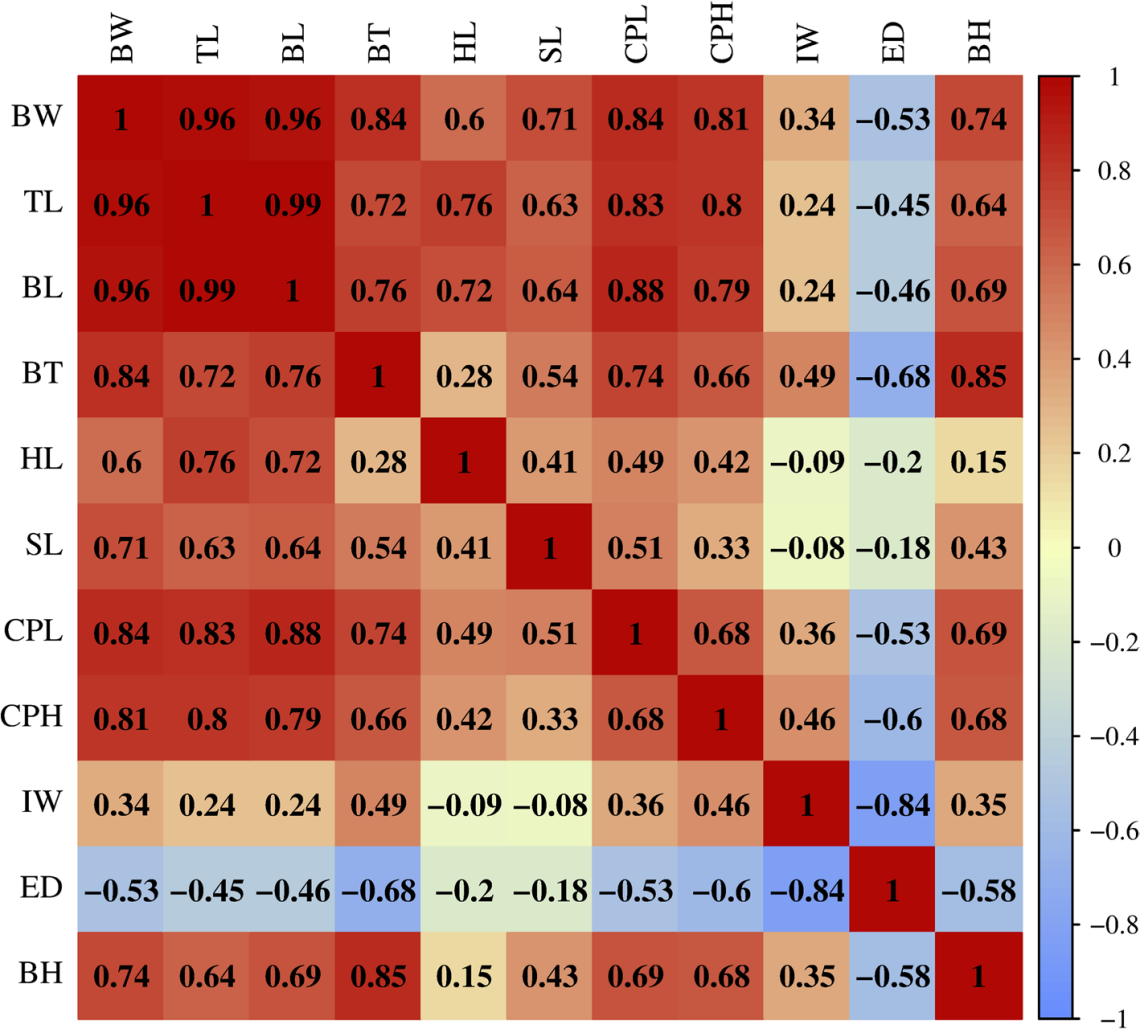
Heatmap display of Pearson correlation coefficients (r) between differet growth-related traits. Red: higher correlation, blue: lower correlation. BW, Body weight; TL, Total length; BL, Body length; BT, Body thickness; HL, Head length; SL, Snout length; CPL, Caudal peduncle length; CPH, Caudal peduncle heigh; IW, Interorbital width. ED, Eye diameter; BH, Body height

The average values of growth-related traits, including body weight (BW), total length (TL), body length (BL), body thickness (BT), head length (HL), snout length (SL), caudal peduncle length (CPL), caudal peduncle height (CPH), interorbital width (IW), eye diameter (ED) and body height (BH) for153 *T. fasciatus* offspring are shown in Table S1. All the growth-related traits of the individuals were normally distributed, with characteristics of continuous variation. The highest correlation (*r* = 0.970) was observed between BL and TL, and the lowest correlation (*r* = 0.322) was observed between ED and IW (Fig. 1 and Table S2).

### Genome resequencing and genome-wide SNP discovery

Raw reads were obtained from two *T. fasciatus* parents and their 153 full offspring by sequencing on the Illumina HiSeqXten platform. Subsequent to filtering low-quality reads, 141.998 × 10^7^ clean reads were obtained (Table S3). The total number of clean reads for the 153 F1 individuals was 13,397,707,886, with an average of 8,813,867 clean reads per F1 individual (GC% of 45.6). The alignment of the clean reads to the reference genome revealed that the proportion of mapped reads was 98.47%. The 153 offspring had an average genome coverage of 96.54 ~ 98.73% (mean 98.46%) and a depth of 3.49 ~ 7.39 × (mean 5.66 ×). The average genome coverage of the two parents was 98.63%, and the average depth was 25.33 × .

### SNP discovery and genotyping

The obtained BAM files were SNP detected and filtered using the GATK protocol, resulting in a total of 185,990,380 SNPs (Table S4). After examination for deviation from Mendelian segregation, 589,463 high-quality SNPs were finally obtained and merged into 5520 polymorphic markers for linkage analysis. These SNPs were classified into three categories: maternal heterozygous (2657 SNPs), paternal heterozygous (2409 SNPs), and heterozygous in both (454 SNPs). All the SNPs are listed in Table S4.

### Construction of the high-resolution linkage map

**Fig. 2:**
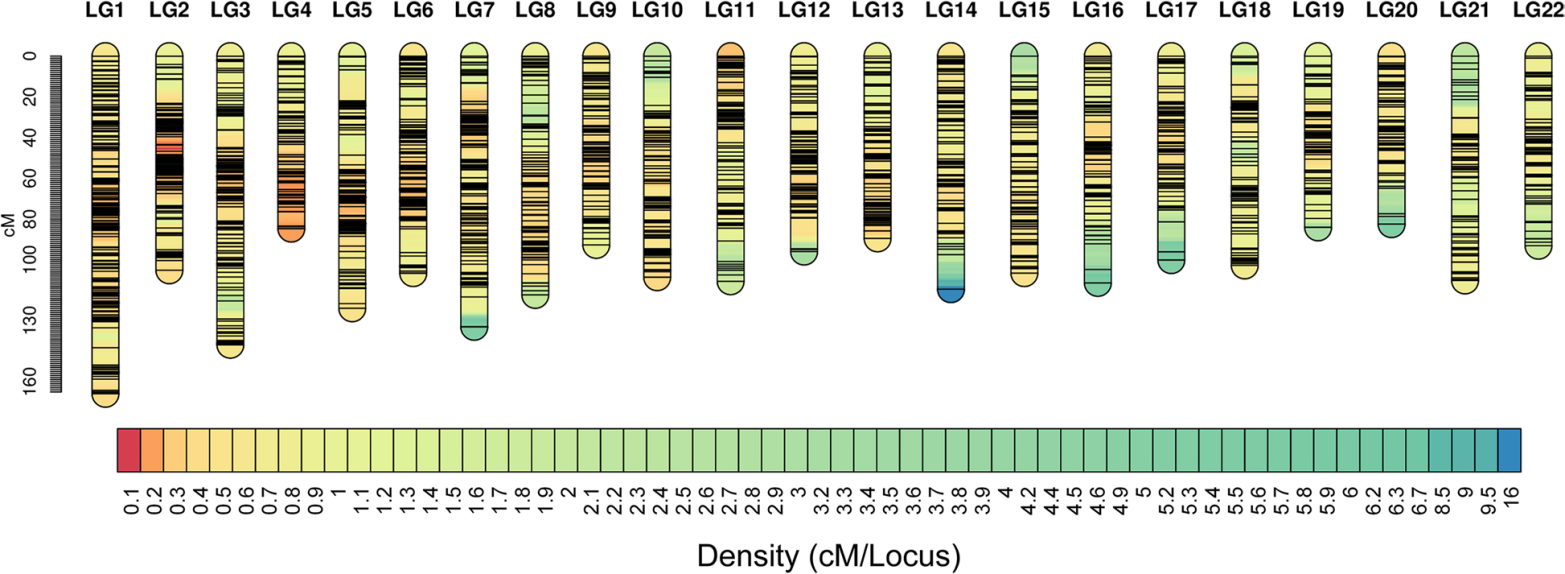
SNP-based linkage map for *T*. *fasciatus* (More details are available in web version.)

Using the Lep-Map3 software package with an LOD threshold of 11.0, 4891 markers (88.6% of all 5520 polymorphic markers) were successfully mapped onto 22 LGs, consistent with the haploid chromosome number (2n = 44) in *T. fasciatus*. The total length of this map was 2381.353 cM with an average interlocus distance of 0.535 cM. The genetic length of each LG ranged from 82.475 cM (LG20) to 165.872 cM (LG1) with interlocus distances of 0.29 to 0.88 cM (Table 1, Fig. 2).

**Table 1:** Summary of statistics for linkage map in *T. fasciatus*

Group ID	Marker count	Total cM	Average cM	Max gap
LG1	464	165.873	0.36	9.708
LG2	332	105.163	0.32	11.732
LG3	320	141.786	0.44	8.92
LG4	294	84.732	0.29	7.005
LG5	297	123.939	0.42	15.152
LG6	284	106.580	0.38	11.732
LG7	280	133.031	0.48	14.712
LG8	243	117.313	0.48	5.889
LG9	220	92.727	0.42	4.798
LG10	221	108.565	0.49	13.842
LG11	205	110.578	0.54	10.106
LG12	198	96.022	0.48	15.152
LG13	191	89.524	0.47	4.799
LG14	190	114.473	0.60	16.955
LG15	177	106.648	0.60	9.313
LG16	170	111.435	0.66	12.566
LG17	170	100.228	0.59	14.712
LG18	137	102.849	0.75	10.106
LG19	135	84.118	0.62	6.258
LG20	131	82.475	0.63	12.565
LG21	126	110.242	0.87	8.921
LG22	106	93.052	0.88	9.312
Total	4891	2381.353	-	-

### QTL mapping for growth and cold tolerance traits

**Fig. 3:**
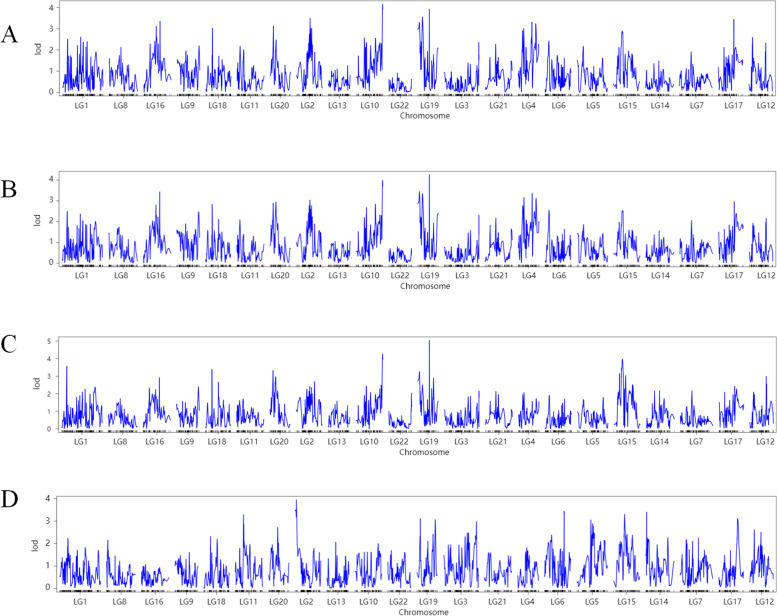
LOD distributions along the linkage map for the whole genome scan for (**A**) body length (**B**) total length and (**C**) body weight (**D**) cold tolerance in *T. fasciatus*. The lines denote the LOD thresholds for estimated significant QTL (More details are available in web version.)

In this study, we conducted 4 QTLs localization analyses, including 3 growth traits and 1 cold-tolerance-related trait. The genome-wide significance thresholds of QTLs were 3.461, 3.761, 4.347 and 2.725 for BL, TL, BW and CT respectively (Table 2). The results of the genetic mapping QTL tests for the four traits are as follows (Fig. 3). By constructing a genetic linkage map, we completed the QTL localization for the growth traits of *T. fasciatus*, and a total of 19 QTLs related to BW, TL and BL were detected in LG1, 2, 4, 6, 10, 15, 16, 17, 18, 19 and 20. Moreover, BW and BL had the same confidence interval on LG2, 10, 15, 16, 18, 19 and 20. A total of 11 QTLs associated with cold tolerance were identified, each scattered on different LGs, namely LG2, 3, 5, 6, 11, 12, 14, 15, 17, 19 and 20.

**Table 2:** Detected QTLs associated with growth traits and cold tolerance traits in *Takifugu fasciatus*

Trait	LG	Position (cM)	Locus	LOD	Pval
BL	LG1	74.418	*scaffold_11*	2.607	1
	LG2	55.605	*scaffold_15*	3.492	0.792
	LG4	56.471	*scaffold_1*	3.313	0.889
	LG10	108	*scaffold_17*	4.155	0.37
	LG15	35.025	*scaffold_5*	2.895	0.994
	LG16	66	*scaffold_11*	3.353	0.866
	LG18	26.578	*scaffold_13*	3.033	0.977
	LG19	47.286	*scaffold_19*	3.924	0.495
	LG20	12.693	*scaffold_14*	3.129	0.959
TL	LG2	55.605	*scaffold_15*	3.034	0.967
	LG4	56.471	*scaffold_1*	3.353	0.835
	LG6	16	*scaffold_3*	2.551	1
	LG10	108	*scaffold_17*	3.992	0.396
	LG15	35	*scaffold_5*	2.526	1
	LG16	66	*scaffold_11*	3.434	0.778
	LG17	63.568	*scaffold_16*	2.956	0.982
	LG18	26.578	*scaffold_13*	2.833	0.994
	LG19	47.286	*scaffold_19*	4.256	0.27
	LG20	23.946	*scaffold_14*	2.946	0.983
BW	LG1	21.58	*scaffold_1*	3.562	0.702
	LG2	74.679	*scaffold_15*	2.691	0.999
	LG10	108	*scaffold_17*	4.261	0.307
	LG12	67.275	*scaffold_9*	2.984	0.969
	LG15	34	*scaffold_5*	3.966	0.465
	LG16	66	*scaffold_11*	2.908	0.983
	LG18	27	*scaffold_13*	3.378	0.838
	LG19	47.286	*scaffold_19*	5.035	0.083
	LG20	12.693	*scaffold_14*	3.317	0.869
CT	LG2	5.736	*scaffold_15*	3.951	0.459
	LG3	133.833	*scaffold_2*	2.97	0.991
	LG5	56.41	*scaffold_4*	3.053	0.976
	LG6	79	*scaffold_3*	3.443	0.812
	LG11	33	*scaffold_13*	3.286	0.903
	LG12	19	*scaffold_9*	2.61	1
	LG14	3.287	*scaffold_1*	3.402	0.837
	LG15	45	*scaffold_6*	3.309	0.891
	LG17	76	*scaffold_8*	3.11	0.965
	LG19	14.464	*scaffold_19*	3.108	0.965
	LG20	36.683	*scaffold_14*	2.719	0.999

BL, TL, BW, and CT represent body length, total length, body weight and cold tolerance respectively. LG, LOD, GW and Pval represents the confidence level, the smaller the value, the more significant the Peak

### Potential candidate gene screening

**Fig. 4:**
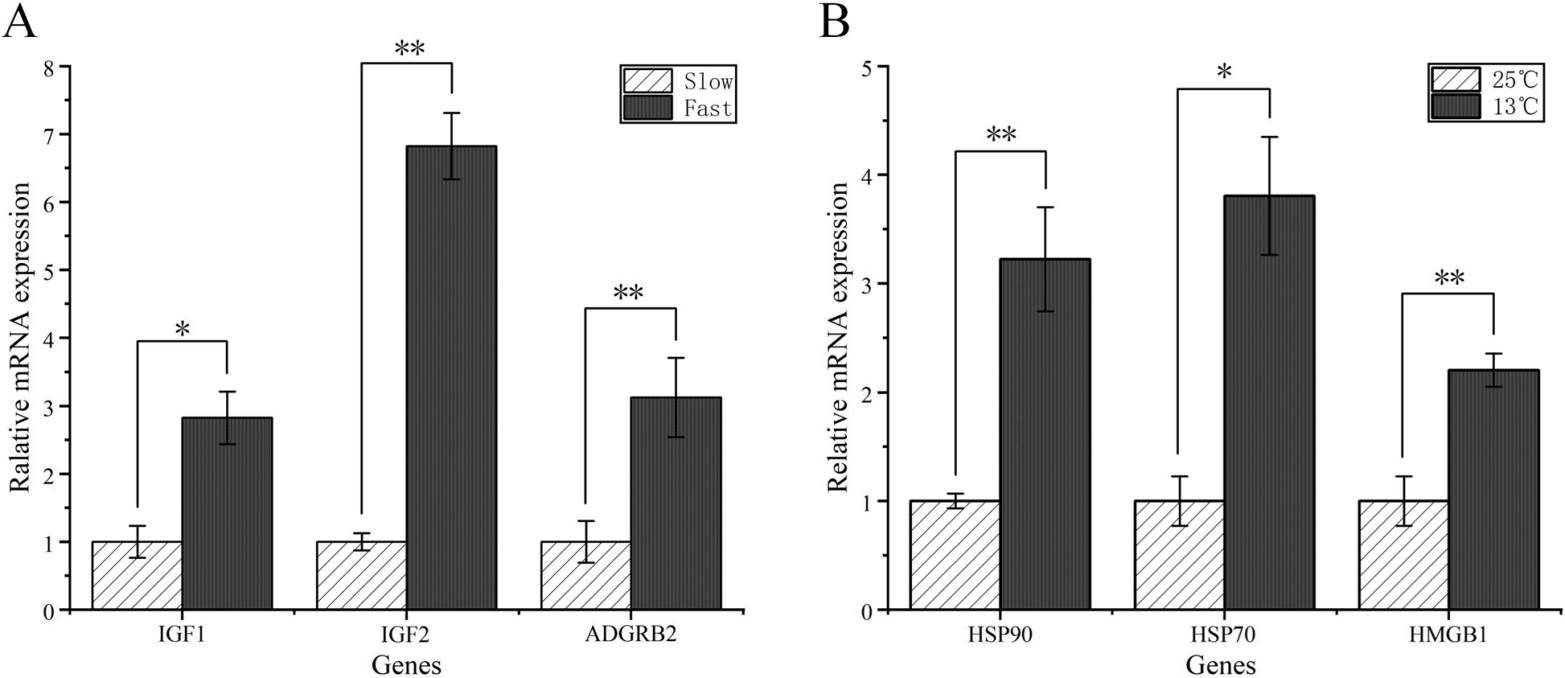
**A** mRNA expression of candidate genes for growth of *T. fasciatus* with different growth rates. **B** mRNA expression of cold-tolerant candidate genes in *T. fasciatus* at different temperatures. *and **denote significance level of *P* < 0.05 and 0.01, respectively

Based on the annotated information for the *T. fasciatus* genome, a large number of candidate genes were identified at QTLs related to growth and cold tolerance, that play important roles in the genetic regulation of growth and development, cell proliferation, immunity and energy metabolism (Table S5). Six candidate genes were selected and validated at the transcriptional level. The qRT-PCR results shown in Fig. 4 revealed that the expression of three cold tolerance-related genes (*HSP90*, *HSP70* and *HMGB1*) was significantly higher in muscle treated at 13 °C than at 25 °C (*P* < 0.05). Three growth-related candidate genes (*IGF1*, *IGF2* and *ADGRB2*), were significantly more highly expressed in fast-growing than in slow-growing *T. fasciatus* (*P* < 0.05) (Fig. 4).

## Discussion

High density genetic linkage mapping is an important prerequisite for genetic profiling of the location of genes or QTLs associated with target traits [18, 19]. Linkage mapping has been applied to many aquatic species, and growth and cold tolerance are economically important traits for *T. fasciatus*. To date, no QTL analysis has been reported for growth or cold tolerance traits in *T. fasciatus*. Therefore, the linkage mapping and QTL analysis in this study will provide a powerful tool for further research and breeding.

### High-resolution genetic map

With technological development, genetic mapping has been widely applied to aquatic animal breeding [20]. To date, genetic linkage maps have been constructed for many aquatic animals, such as tiger puffer (*Takifugu rubripes*) [21] and Chinese giant salamander (*Andrias davidianus*) [22]. The selection of a mapping population is a prerequisite for constructing a genetic linkage map. Different mapping family types, including recombined inbred lines (RIL), haploid (HAP) and doubled haploid (DH), subgeneration (F1), subgeneration (F2) and back crossing (BC), have advantages and disadvantages [23]. At present, the F1 mapping family is used to construct genetic linkage maps for most aquatic species. In this study, 1,462,637 high-quality SNP markers were screened, and 4,891 SNP markers were obtained for the construction of the F1 *T. fasciatus* genetic linkage map. The total length was 2281.35 cM, with an average length of 108.243 cM. The average genetic distance of the markers was 0.535 cM, indicating that the marker genetic map constructed in this study was of high density, good quality and uniform marker distribution, and the map contained 22 LGs, which was consistent with the chromosome number of *T. fasciatus*, indicating that the map had high credibility. As far as we know.This map is the first one to be produced for *T. fasciatus*, and it is the highest linkage genetic map among all genetic maps for the *Takifugu* genus. Contrary to the previous genetic map established by Shi [4], which used a full-sib offspring of *Takifugu bimaculatus*, the total length of their map was 2039.74 cM, and the number of markers was much 1079. The genetic linkage map we constructed is superior to their, which reduced the average genetic distance between neighboring markers from 1.13 cM to 0.535 cM. In the present study, genetic maps of high density and high resolution were constructed, providing an important tool for future fine mapping of MAS.

### QTL localization and candidate gene identification of growth-related traits

Growth traits are key to the genetic improvement of economically farmed fish, and many fish species, such as *Ctenopharyngodon idellus* [24] and *Pelteobagrus vachelli* [25], have been targeted for growth-related QTL based on the construction of genetic linkage maps. In this study, QTLs for the growth traits of *T. fasciatus* were analysed for the first time based on a constructed genetic linkage map. QTL analysis is helpful for identifying trait-related linkage markers and predicting candidate genes. Current studies have shown that fish growth traits are mainly regulated by microefficient polygenes. Growth phenotypic traits were not previously isolated but were interlinked. To facilitate subsequent analysis of QTL localization results, 11 growth phenotypic traits (BW, TL, BL, BT, HL, SL, CPL, CPH, IW, ED and BH) were subjected to correlation coefficient analysis in this study, and the results showed strong correlations among BW, TL, and BL. A total of 19 QTLs related to BW, TL and BL were detected in 11 LGs, which indicated that growth traits were influenced by multiple QTLs and genes. For example, Wang et al. [26] identified QTLs for BW in two LGs of *Lates calcarife*. Liu et al. [27] identified QTLs for BW in *Larimichthys polyactis* on 3 different LGs. Moreover, BW and BL in this study had the same confidence interval on LG2, 10, 15, 16, 18, 19 and 20, which confirms their relatively high correlation coefficients. This high correlation coefficient has been found in other aquaculture species (*e.g.*) [21, 27, 28].

The study of candidate genes related to *T. fasciatus* growth traits can help to improve breeding efficiency of the species. In this study, three candidate genes, *IGF1* (insulin-like growth Factor 1), *IGF2* (insulin-like growth Factor 2) and *ADGRB2* (adhesion g protein-coupled receptor B2), were screened from growth-related QTLs and qRT-PCR experiments were performed. The results showed that the three genes showed different levels of expression in fast-growing fish, indicating that these genes play a potential regulatory function in the rapid growth of *T. fasciatus*. *IGF1* and *IGF2* are both considered to be important receptor synthesis mediators that exert biological effects in growth hormone receptor synthesis and belong to a class of growth hormone receptor mediators where insulin plays a decisive role in the growth of tissue cells in the immediate postnatal period in mammals [29]. IGF1 has a specific growth hormone-binding receptor protein (IGFBP) and a growth hormone-binding receptor protein with a specific IGF receptor, an endocrine growth hormone and an endocrine and paracrine growth regulator of its own [30, 31]. The role of IGF1 in growth has been relatively well studied, with the main focus on livestock [32], and relatively little research and reporting in aquaculture. Davis et al. studied polymorphisms in the IGF1 gene and found that this gene polymorphism had a significant effect on weight gain and milk yield growth in cows [33]. In the present study, the expression of *IGF1* and *IGF2* was elevated in fast-growing fish, and we speculate that they play a role in promoting the growth of *T. fasciatus*. ADGRB is an evolutionarily ancient subgroup of the GPCR (adhesion G protein-coupled receptors) superfamily, and plays a key molecular switch role in many important physiological processes in organisms [34], such as brain development, neurodevelopment, angiogenesis, water and salt regulation, inflammation and cells. ADGRB2 was significantly expressed in fast growing *T. fasciatus*, and we speculate that ADGRB2 promotes the fast growth of *T. fasciatus*. However, research on this gene is still relatively scarce and further in-depth studies can be conducted in the future.

### QTL localization and candidate gene identification of cold tolerance traits

*T. fasciatus* is a warm water migratory economic fish, and breeding cold-tolerant species can promote healthy development of the fish farming industry. In addition, cold tolerance is considered to be an important trait in cultured fish, and is considered to be a qualitative trait controlled by multiple genes [1, 35]. In this study, a total of 11 QTLs associated with cold tolerance were identified, each scattered on different LGs, namely LG2, 3, 5, 6, 11, 12, 14, 15, 17, 19 and 20, suggesting that cold tolerance traits may be controlled by multiple genes on multiple chromosomes. Liu et al. [21] constructed a high-density genetic linkage map for *Takifugu rubripes* (a close relative of *T. fasciatus*) and a QTL map of cold resistance traits, and a total of eight QTLs related to cold resistance were detected in LGs 5, 7, 10, 15, 16, and 22. This result could also suggest that the cold tolerance trait may be controlled by multiple genes on multiple chromosomes. Although there are common chromosomes between growth-related traits and cold tolerance traits, they are not in the same location, suggesting that growth and cold tolerance traits are not well correlated and that finding a gene that is correlated with both growth and cold tolerance may be difficult. In the future, we will develop synergistic gene for the growth and low-temperature tolerance of *T. fasciatus.*

In this study, three genes associated with low-temperature tolerance, *HSP90* (Heat shock protein 90), *HSP70* (Heat shock protein 70) and *HMGB1* (High mobility group Box 1), were screened from the low-temperature tolerance-related QTL for expression analysis under low-temperature stress. All three genes showed different levels of expression in the tissues under low temperature stress, indicating that these genes play a potential regulatory function in the low temperature adaptation of *T. fasciatus*. HSP90 plays a crucial role in protein folding, cell signalling and protein degradation. HSP90 can be regulated by a variety of environmental stressors, such as heat shock, heavy metals and pathogenic infections [36, 37]. Peng et al. found that kaluga (*Huso dauricus*) showed significant changes in *HSP90* expression in its muscle, gill and liver at low temperatures [38]. The results of Li et al. [39] also showed that HSP90 is significantly expressed at low temperatures in *Oryzias melastigma* larvae. Similarly, oue results show that *HSP90* is significantly expressed in the muscles of *T. fasciatus* at low temperatures. HSP70 is a kind of highly conservative protein that is rapidly synthesized under stress stress [40]. Such stress response can provide protection, tolerance, and cross tolerance; can mitigate stress caused by abnormal or denatured proteins; can have the effect of activating other cell genes; and can inhibit cell apoptosis caused by ATP loss [41, 42]. Liu et al. [43] found that low-temperature stress increased *HSP70* levels in *Puntius tetrazona*, suggesting that the expression of the molecular chaperone *HSP70* in *P. tetrazon*a may play a key role in the response to acute cold stress. In this study, the qRT-PCR results showed that the expression of *HSP70* in muscle showed an increasing trend in the low temperature group. HMGB1 is an abundant and charge-rich nuclear protein that plays an important function in organisms [44, 45]. In this study, the expression of HMGB1 in the muscle of *T. fasciatus* showed an increasing trend in the low temperature group. Therefore, the increased HMGB 1 expression in the muscle of *T. fasciatus* during cold stress may be a response of the body to protect the fish from low-temperature damage.

## Conclusion

A high-density genetic map of *T. fasciatus* was constructed using the WGR method. The map contained 4891 bin markers, which were distributed in 22 LGs, consistent with the number of chromosomes in *T. fasciatus* (2n = 44). The total length of the genetic linkage map was 2831.353 cM, and the average density was 0.535 cM, which is the highest density genetic linkage map of *T. fasciatus* to date. Furthermore, we successfully identified QTLs related to growth and cold tolerance traits using this map, and identified three candidate genes (*IGF1*, *IGF2* and *ADGRB2*) related to growth traits, and three genes (*HSP90*, *HSP70* and *HMGB1*) related to cold tolerance traits. This study will provide valuable data for the subsequent molecular breeding of *T. fasciatus* and will promote the healthy development of the *T. fasciatus* breeding industry.

## Material and methods

### Mapping population and phenotypic data

The male and female parents were chosen from a cultured population from the ZhongYang Group in Nantong, Jiangsu Province, China. The F1 generation was cultured in the same environment and fed artificial compound feed twice daily. After 5 months of culturing the F1 generation, a total of 153 individuals were randomly collected and maintained in a 150 L tank with flow-through water for one week before trait measurement. Then, cooling was applied at 1 °C per hour by the automatic temperature control equipment of the recirculating aquaculture system. The cooling rate was slowed when it reached 13 °C, and subsequent cooling was carried out by adding ice to the circulation system (not directly to the experimental tank). It took 5.5 h to reduce the water temperature from 13 °C to 6.8 °C. Intolerance to cold temperatures was judged by a fish sinking to the bottom of the tank, indicating a loss of equilibrium, and the time at which loss of equilibrium occurred was recorded for each fish. The fish were killed by dissection after mild anaesthetization in a eugenol bath (1:10,000). while growth traits such as body weight (BW), body length (BL) and total length (TL) were measured. The duration from the onset of cooling to the state of cold shock was defined as a trait of cold tolerance (CT). After the traits were assessed, a portion of the caudal fin of each fish was cut and placed in cryovials and stored at -80 °C.

### Library construction and sequencing

DNA was extracted from the caudal fins of the 155 T*. fasciatus* (153 F1 + 2 parents) using an Animal Genomic DNA Kit from Beijing Biotec Biotechnology Co. DNA quality, concentration, and integrity were evaluated using a NanoDrop-2000 spectrophotometer and 0.8% gel electrophoresis spectrophotometer. The qualified DNA samples were randomly interrupted to screen qualified DNA fragments of appropriate size. The total amount of DNA required to detect a single sample library was greater than or equal to 10 μg.

The DNA samples were randomly broken into fragments of 350 bp in length by a Covaris fragmentation machine. The library was constructed using the TruSeq Library Construction Kit, using the recommended reagents and consumables, and the entire library was prepared by end repair, addition of ployA tails, addition of sequencing connectors, purification and PCR amplification. After the library was constructed, the library was initially quantified using Qubit 2.0 and was diluted to 1 ng/μl, and then the insert size of the library was checked using Agilent 2100. The libraries were then accurately quantified using qRT-PCR (quantitative real-time PCR) (> 2 nM) to ensure library quality.

### SNP discovery and genotyping

The original library was first filtered as follows: 1) reads containing splice sequences had to be filtered out; 2) bases with a contiguous mass of less than 20 at each end of the sequenced read were removed; 3) when the final length of the sequenced read was less than 50 bp, the read was removed; 4) and only paired reads were retained.

The resulting clean reads from each individual were aligned against the *T. fasciatus* reference genome (NCBI database accession number: PRJNA449558) [8] using Burrows‒Wheeler Aligner (BWA) software [46] (settings: mem -t 4 -k 32 -M -R). The reads were aligned to the reference genome and deduplicates of the PCR repeats (duplicates reads) were conducted using Picard software. The deduplicated data were then counted for alignment rate, coverage and sequencing depth. Finally, the sample data were filtered and corrected, and the population data were tested for variation and filtered using GATK software (− -algo Haplotyper –emit_conf = 10 –call_conf = 30) to obtain high confidence SNPs. The annotation of the SNP sites detected in the samples was carried out using ANNOVAR software.

The screening and filtering criteria for polymorphic markers were as follows: 1) Sequencing depth of no less than 15 and no more than 1000 for any one parent and SNP quality of no less than 10; 2) Sequencing depth of no less than 5 and no more than 1000 for any one locus per offspring; SNP quality of no less than 10. If the filtering criteria were not met, the genotype was judged to be missing to ensure the accuracy of the genotyping results. The genotyping results are shown in the table below (Table 3).

**Table 3:** Types of polymorphic marker development

Marker Type	First parent genotype	Second parent genotype	Expected segregation ratio	Numbers
hk x hk	hk	hk	1:2:1	320,025
lm x ll	lm	ll	1:1	521,722
nn x np	nn	np	1:1	507,237
Total markers				1,348,984

### SNP identification

The selection of mapping markers was based on the selection of different polymorphic marker types for different population types. For F1 proposed cross populations (CP populations) from heterozygous parental crosses, we retained markers with genotypes (lm x ll, nn x np, hk x hk) for subsequent mapping. Depending on the type of population, the developed markers were screened as follows: 1) Abnormal bases: the offspring typing results may show base types that are not present in the parents; 2) Completeness: screening for markers whose genotypes cover at least 95% of the individuals in the offspring; 3) Chi-square screening: Based on the classification of each type of marker in the CP population, we filtered for polymorphic markers that were severely segregated (*P* value less than 0.05) using a chi-square test.

### High-density genetic linkage map construction

The above filtered SNP loci were used for genetic mapping construction using the Lep-Map3 software package [47] in the following steps. 1) High quality SNPs were clustered to linkage groups by the SeperateChromosomes2 module, using pairwise likelihood of odd (LOD) scores (lodLimit parameter) for the delineation of chain clusters. 2) The chain clusters were encrypted with the JoinSingles2All module, lowering the LOD threshold for dividing the chain clusters by recalculating the LOD values of markers not divided into chain clusters and markers already divided into chain clusters, and again assigning these undivided markers to the appropriate chain clusters. 3) Using OrderMarkers2, the algorithm of multipoint maximum maximum likelihood (MML) was used for ranking the map.

### QTL mapping for growth and cold tolerance traits

QTL localization analysis was performed using the QTL localization R package R/qtl (step = 1). For the analysis in this project, we chose 1 cM as the size of the scan interval and performed a stepwise scan across the genetic map to identify QTLs. We used LOD = 2.5 as the threshold for QTL detection in this project. For all QTL regions that exceeded the threshold, we first located where the peak (LOD maximum) of this significant QTL was located. Candidate genes controlling the trait are usually located near the QTL peak. By convention, the interval corresponding to the QTL interval after decreasing the LOD maximum by 2 for each QTL is generally taken as the interval where the potential candidate genes are located, and this interval is called the 2-LOD confidence interval.

### Identification of potential candidate genes

To verify the accuracy of the candidate genes, we acquired additional samples for experimental processing. For the validation of growth candidate genes, we raised juvenile *T. fasciatus* in the same environment. After three months of breeding, we selected individuals with different growth rates and divided them into fast and slow growth groups of six fish each. The weight of individuals in the fast-growing group was 120 ± 10 g, and the weight of individuals in the slow-growing group was 80 ± 10 g. For cold tolerance candidate gene validation, the experiment was set up in two groups of 13 °C and 25 °C (control), with forty-five fish in each group and three replicates of fifteen fish each. On the basis of our previous study [48], the water temperature was reduced from 25 °C to 13 °C at a rate of 0.85 °C/1 h to prevent fish stress mortality. After 24 h at 13℃, six fish with normal motility were randomly selected from each temperature group. Muscle samples were collected and stored in a refrigerator at -80 °C.

Total RNA was extracted from the samples using the RNAsimple Total RNA kit, and the RNA purity was checked using 1% agarose gel electrophoresis. The first strand of cDNA was synthesized using the FastKing RT kit and placed at -20 °C for subsequent experiments. The primers were designed using Primer 5.0 software. The primers were synthesized by Bioengineering (Shanghai) Co. and tested by PCR. The primer sequences are shown in Table 4.

**Table 4:** Primers used in this study

Primer	Primer sequence (5′ ~ 3′)
*IGF1* F	GCTCCCGCCAAGACGAACAAG
*IGF1* R	TTGTCCGCTTTGTGCCCTGTG
*IGF2* F	AGCCCAAGCCGCCTATCTGTC
*IGF2* R	CTTCCTCTGCCACACCTCGTATTTG
*ADGRB2* F	AGTGACCAGCCCAGTTCAGAGG
*ADGRB2* R	GCCGCAGTTCCAGCAGTTCC
*HSP90* F	AAGGAGGATGAGGAGCGAGATACTG
*HSP90* R	AGCACACTATTCCAGGGCAGTTTC
*HSP70* F	CAAGAGCGTCCAGCCAATCAGAG
*HSP70* R	ACTCGTCCACCTCCTTCACCAAG
*HMGB1* F	GCCGTCCGCATTCTTCCTCTTC
*HMGB2* R	GCCGTGTCGCCAATCGTGAG
*β-actin* F	CCAGAAAGACAGCTACGTTGG
*β-actin* R	GCAACTCTCAGCTCGTTGTAG

The qRT-PCR was performed on an Applied Biosystems StepOnePlus PCR instrument using the reverse transcribed cDNA as the template. Reactions were performed according to the TOROGreen qPCR Master Mix kit (QST-100) manufacturer instructions. The reaction system was 20 µL: Master Mix 10.0 µL, RNase-Free water 7.2 µL, upstream and downstream primers 0.4 µL each, template 2 µL. The reaction procedure was as follows: predenaturation at 95 °C for 60 s; denaturation at 95 °C for 10 s; and annealing at 60 °C for 30 s, 40 cycles. The mean expression and variance were calculated using the 2^−ΔΔCt^ method.

### Statistical analysis

One-way ANOVA was performed using SPSS 18.0 software to test whether the differences between the mean expression levels of the groups were significant (*P* < 0.05 indicates a significant difference). The plots were made with Origin software (Origin 2021).

### Supplementary Information

**Additional file 1: Table S1.** Phenotypic data and time spent out of equilibrium from sequenced populations of *Takifugu fasciatus*.

**Additional file 2: Table S2.** Correlation coefficients for growth traits.

**Additional file 3: Table S3.** Data production of WGR sequencing for each individual.

**Additional file 4: Table S4.** Polymorphism SNP markers and their association sequence information.

**Additional file 5: Table S5.** All potential candidate genes for significant genome-wide QTLs.

## Data Availability

The datasets supporting the conclusions of this article are included within the article and its additional files except the raw sequencing reads that are available on NCBI (BioProject Accession Number: PRJNA449558, https://www.ncbi.nlm.nih.gov/bioproject?term=PRJNA449558&cmd=DetailsSearch).

## Abbreviations

QTL: Quantitative trait locus
MAS: Molecular marker-assisted selection
SNP: Single-nucleotide polymorphism
BW: Body weight
TL: Total length
BL: Body length
BT: Body thickness
HL: Head length
SL: Snout length
CPL: Caudal peduncle length
CPH: Caudal peduncle heigh
IW: Interorbital width
ED: Eye diameter
BH: Body height
